# Supplementary material for: Clinical characteristics and outcomes of patients admitted with COVID-19 at a public-sector hospital over the first two waves of SARS-CoV-2 infection in Harare, Zimbabwe: A prospective cohort study
Source: PLOS Glob Public Health. 2024 Jan 25;4(1):e0001100. doi: 10.1371/journal.pgph.0001100 (PMC10810425; doi:10.1371/journal.pgph.0001100)
Supplement: Attachment — Submitted filename: Response to Reviewers.docx [file pgph.0001100.s002.docx]

**BIOMEDICAL RESEARCH AND**

**TRAINING INSTITUTE**

**10 Seagrave Road**

**Cnr S. Nujoma St & Seagrave Rd**

**Avondale**

**Harare**

**P.O. Box CY 1753**

**Causeway, Harare, Zimbabwe**

**Tel: 263 4 336691; 335641; 333091**

**Fax: 263 4 333464**

**E-Mail:** [**snyauzame@brti.co.zw**](mailto:snyauzame@brti.co.zw)

**Website: http://www.brti.co.zw**

A Non-Profit Organization Promoting Health Research for Development in Southern Africa

**BRTI**

The Editor

PLOS Global Public Health

21^st^ December 2022

Dear Editor,

Thank you for the constructive, detailed feedback and comments on our manuscript titled “*Clinical characteristics and outcomes of patients admitted with COVID-19 at a public-sector hospital over the first two waves of SARS-CoV-2 infection in Harare, Zimbabwe: A Prospective cohort study”* (PGPH-D-22-0014). Please find a point-by-point response to the reviewers' comments below:

**Data availability**

After discussion with our data team and repository manager as informed consent was not obtained from participants, public deposition of the dataset would breach the Medical Research Council of Zimbabwe (MRCZ) requirements for data from a public hospital. Interested parties should contact the Parirenyatwa Group of Hospitals regarding access to the data. We therefore request an exemption from the editors for the reason mentioned above.

**Reviewer Two**

‘’The authors addressed some reviewers comments on this revised manuscript version. Although the manuscript improved, some limitations still need to be addressed.’’

1) Since the authors use mortality as main outcome. I recommend to use a more accurate statistical test (eg, Cox regression) to verify consistency. It could be performed as main analysis or as a complementary sensitivity analysis.

- Thank you for highlighting this. We performed Cox regression as a complementary sensitivity analysis. We have added the following in the statistical analysis section: ‘’As a sensitivity analysis, Cox regression with robust standard errors was used to estimate the mortality hazard ratio in patients who stayed more than 1 night, adjusting for the same variables used in the Poisson multivariate regression.’’
- We have also added in Table 4 (Factors associated with mortality in participants who stayed in the ward for more than 1 night, using multivariate Cox regression models) adding the following into the manuscript ‘’ Table 4 shows the Cox regression for mortality hazard ratio using the same covariates. The association of age category with mortality hazard was not statistically significant on a Wald test. Otherwise, results were comparable to the results of the Poisson regression.’’

2) There are some inconsistencies in manuscript writing. In this sense, I might suggest to edit the manuscript for grammar and style.

- We have been through and reviewed the manuscript to ensure there are no further grammatical errors and to ensure a consistent style throughout. We have made some minor changes to sentence structure to make a more cohesive, easy-flowing paper.

**Reviewer Three**

1) The discussion around the resource limitations in which this service operated are key to be able to lobby for additional health care resources in the future.

   Specifically the statement: "After 1st November 2020, lack of resources was felt to have contributed to 57 (35.4%) of 161 deaths.’’ I feel should be qualified to be "After 1st November 2020, lack of resources was felt by the treating clinical team to have contributed to 57 (35.4%) of 161 deaths."

- We agree the suggested wording more accurately describes it therefore we have changed the statement as guided by the reviewer.

2) Rest of the suggestions are minor and stylistic. See attached comments in PDF. The paper reads well to me and as with all such studies the context is important, and I feel adequately described given the challenges of healthcare delivery in Africa.

- We thank this reviewer for going through the paper and suggesting changes. We have reviewed them all and have made the appropriate changes suggested.

We look forward to hearing from you

Yours sincerely

Dr Arun Fryatt, on behalf of authors
