# Supplementary material for: Clinical characteristics and outcomes of patients admitted with COVID-19 at a public-sector hospital over the first two waves of SARS-CoV-2 infection in Harare, Zimbabwe: A prospective cohort study
Source: PLOS Glob Public Health. 2024 Jan 25;4(1):e0001100. doi: 10.1371/journal.pgph.0001100 (PMC10810425; doi:10.1371/journal.pgph.0001100)
Supplement: Attachment — Submitted filename: Response to Reviewers.docx [file pgph.0001100.s004.docx]

**BIOMEDICAL RESEARCH AND**

**TRAINING INSTITUTE**

**10 Seagrave Road**

**Cnr S. Nujoma St & Seagrave Rd**

**Avondale**

**Harare**

**P.O. Box CY 1753**

**Causeway, Harare, Zimbabwe**

**Tel: 263 4 336691; 335641; 333091**

**Fax: 263 4 333464**

**E-Mail: [snyauzame@brti.co.zw](mailto:snyauzame@brti.co.zw)**

**Website: http://www.brti.co.zw**

A Non-Profit Organization Promoting Health Research for Development in Southern Africa

**BRTI**

The Editor

PLOS Global Public Health

21^st^ September 2023

Dear Editor,

Thank you for the constructive, detailed feedback and comments on our manuscript titled “*Clinical characteristics and outcomes of patients admitted with COVID-19 at a public-sector hospital over the first two waves of SARS-CoV-2 infection in Harare, Zimbabwe: A Prospective cohort study”* (PGPH-D-22-0014). Please find a point-by-point response to the reviewers' comments, suggestions, and/or corrections below.

**Academic Editor comments**

- ‘Abstract- replace data was to "data were"
- ‘This study looks to add..., replace with this study aimed to.... or the aim of this study was to....’
- ‘Line 90-91, a sentence reads "The time between ...was defined as first wave. Add Zimbabwe after first wave as this definition is based on Zimbabwe context and may not apply in other contexts.’
- ‘Authors should ensure consistency in font size. E.g., check table 4 heading.’
- ‘Write ICU in full the first time you use it - line 134’
- ‘Discussion: Check for typos and punctuations. e.g. paragraph 3, correct punctuations before a sentence that reads "likely reasons for late presentation." Also, add a punctuation on "of note, 21% of deaths...."’

All the suggestions above were changed in the manuscript.


**Reviewer 4**

**General Comments**

- ‘All variables need definitions.’
  - All variables have been defined. This is discussed in ‘Methodology’ below in more detail.
- ‘Keywords are too many. They should be reduced in number.’
  - Number of keywords reduced.

**Abstract**

- Line 29 suggested changes made. ‘‘…. the association between sociodemographic and clinical characteristics and mortality ….’’.
- Line 37-38 suggested changes made. “…mortality was higher in patients with comorbidity compared to those without any comorbidity…”

**Introduction**

- ‘The style of in-text citations brackets should be consistent. Please, edit on line 71 according to the whole text?’
  - Edit completed.

**Methodology**

- Regression methods is fitting with the type of data Variables; steps have been followed. However, none has been said, in the method section, about how the authors tried managing potential over-dispersion or missing of data. This should be said clearly.’
  - Thank you for this comment. We have investigated and found evidence of overdispersion, so we have used a negative binomial model instead of the Poisson regression. There is no substantial change to the results. Nothing was done to manage missing data.
- The manuscript needs clear definitions of variables. I suggest variable definitions for better understanding of the manuscript. For illustration, elsewhere nutrition status of participants should be defined and measures for assessment described. Also, dependent variable and independent variables should be clearly mentioned in the methods section.
  - We have added the following into our methods (Lines 138-141) ‘The dependent variable was mortality. The independent variables were age, sex, location, oxygen saturation, weight category, haemoglobin (gram/decilitre), white blood cells (10^9^/litre), creatinine (micromol/litre), any comorbidity, and pregnancy.’
  - All other variables are described. For the example of nutrition status, it is described in lines 131-133 and called ‘weight category’ in the tables. ‘A considerable number of patients were too unwell for measurement of height and weight. Therefore, weight categories were based on a visual assessment by the clinical team.’
- Line 140: I suggest the authors to insert this relevant citation for the Poisson regression with robust errors (Cameron, A. C. and Trivedi, P. K. (2009). Micro econometrics Using Stata. College Station, TX: Stata Press.)
  - We are no longer using Poisson regression, so the citation is not needed.

**Results**

- Table 2: the total values lines ‘Any complications and ‘Dexamethasone’ need some editing. The authors can, please, check for and edit?
  - Thank you for checking these figures. The table has been reviewed and we have made the following changes: any complication in discharged group 90/340 (26.5%), dexamethasone in total group 401/554 (72.4%).
- Poisson regression in Table 3. Category age under twenty years should be deleted in that sense this makes a bit confusion at reading the table 3 and table 4.
  - Thank you for this comment. We think it could be misleading to remove the category and would prefer to leave it in for completeness.
- Cox regression in Table 4: Category age under twenty years should be deleted in that sense this makes a bit confusion at reading the table 4.
  - Thank you for this comment. We think this could be misleading and we have to present all data that were collected.
- Underweighted individuals were more likely to die compared to overweight. I would like the measure of overweight being defined in the method section for better understating. Also, this contrasting result should better be discussed and provide clarity in the discuss section.
  - As stated above, in Lines 131-133 we explain our methods for classifying the patients into weight categories (‘…weight categories were based on a visual assessment by the clinical team.’). The finding that underweight patients were more likely to die is not surprising and is in line with the literature, so we did not discuss it specifically.
- Limitations of the study. It has been noticed that missing data were recorded in the study. How the authors managed to alleviate the negatives effects of missing data?
  - In our discussion section we discuss the reasons behind the missing data in lines 321-323. ‘The study was conducted during the first two COVID-19 waves within a very busy and understaffed clinical service. Therefore, some data are missing and could not be retrospectively obtained from medical notes.’ These data may not have been missing at random, and we are not able to alleviate the negative effects, so it remains as a limitation.

**References**

- The authors should check for the consistency in the reference list at using the capital letters for the title of the resources. See references numbers 8, 9, 14, 19, 23, 25, 28, 31.
  - References have been reviewed and the appropriate changes have been made for consistency.

**Previous comments and Revisions**

a) At this time disclose the reason for this data restriction and provide a point of contact, preferably an email, for the Parirenyatwa Group of Hospitals in Zimbabwe. Please note that this point of contact cannot be an author of this study.

- As informed consent was not obtained from participants, public deposition of the dataset would breach Medical Research Council of Zimbabwe (MRCZ) requirements for data from a public hospital. Interested parties should contact the Parirenyatwa Group of Hospitals in Zimbabwe through the Lead Health Information Officer Mr Philip Mapurisa - [mutsamaps@gmail.com](mailto:mutsamaps@gmail.com).

**Reviewer One**

***Major***

1. Risk factors associated with death and complications are reasonably performed. However, this study is complicated by the fact that clinical care seems to be uneven across patients. The authors talk about significant limitations in human resources, and as a result, limited advanced treatment (including NIPPV and intubation). However, this is not included in the patient level analysis. As a result, assessing risk for mortality and complications based on clinical characteristics alone is highly limited. and outcomes may actually be explained by differences in clinical care (at least in part). Additionally, the authors state in their conclusion that “evidence-based care was delivered,” yet later note challenges with supervision and limitations to escalate care. This is confusing and requires additional clarification.

- The aim of our study is to describe the clinical characteristics, interventions and outcomes and the risk factors in ***our*** context, with the inherent resource limitations and other issues which we explicitly acknowledge led to uneven and inconsistent care and the observed outcomes. Our risk factors and findings should therefore be considered on the context. We feel this is important because studies from this region and those conducted in routine, “non-specialist” settings, are really limited, and specifically conducted this study, acknowledging the limitations of care, to inform the clinical care needs in such settings. . We entirely agree that some of the differences in outcomes could be explained by the uneven care (the main reason for which was limited human resource capacity) rather than the intrinsic clinical severity, and that is why we have attempted to document and understand when clinical care was not escalated. We now explicitly state this in our Discussion: ‘’ *It is important to note that while evidence-based treatment protocols were implemented, care was delivered amid considerable health system challenges, and may have been uneven across patients, which may, in part, explain the high mortality.*
- In our limitations Section, we now state the following: ‘’*The study assessed risk for mortality and complications based on clinical characteristics and did not include patient level analysis of access to advanced treatments (ICU, HFNO) which means outcomes may, in part, be explained by differences in clinical care.*’’
- The COVID clinical care protocols were based on contemporary evidence, and we aimed to deliver care according to those protocols as much as was possible. However, this was not always possible due to the resource constrained context. We now explain this in the Discussion (see above sentence)

2. The authors focus the majority of their discussion on the issue of limited human resources/capacity. However, this represents just 2 sentence in the results. Further, the way in which attribution of poor outcomes due to limited resources is based on a single provider’s limited documentation leaves many unanswered questions (the reason for lack of escalation is not shown here). I would recommend that the authors present the reasons for inability to escalate care and be clear about this cohort of patients, presenting additional information on their clinical characteristics that may have impacted outcome.

- Thank you for this suggestion We have restructured the Discussion to focus on the clinical care aspects and moved the discussion on limited resources further down to not detract attention from the primary focus of the paper. We agree that further explanation needs to be added in to fully understand the reasons behind escalation and what it means when it was deemed unable to be done. We have further explained the specific reasons when a lack of resources was deemed to cause a poor outcome with the following added in: ‘*’Any patients desaturating (SpO2<88%) on low-flow oxygen (15 Litres via non-rebreather mask) would be offered HFNO, however for 17 patients all 4 machines were in use and they subsequently passed away. All patients who desaturated (SpO2<88%) on HFNO would be escalated to the Intensive Care Unit (ICU) if there was capacity. ICU capacity was limited due to a lack of ICU trained staff (anaesthetists and Intensive Care Nurses). ICU had a maximum capacity of 3 patients.* If a patient required an operation or dialysis but there were no staff available and they subsequently passed away this was counted in lack of theatre or dialysis group*’’*

3. Were all patients admitted to the hospital tested for COVID at the time of admission? Please clarify. This will significantly impact the denominator. Additionally, in the results the authors note that 515 patients “reported” a test for COVID. Was this not abstracted from the chart? Who reported this?

- Not all patients were tested for COVID-19 at the time of admission. Many patients were sent in from other healthcare facilities that had already performed testing for SARS-CoV2 and if they could show evidence of this it was documented as a positive SARS-CoV2 result.
- We used ‘reported’ as some patients were tested at the PGH emergency department but were only admitted later (i.e. became unwell in the following days) and would have to show us their SARS-CoV2 result or be retested. ‘Reported’ was when the patient showed us a printed SARS-CoV2 PCR result.

4. Reframe the groups that are currently referred to as COVID and non-COVID to “admitted due to COVID” and “diagnosed with COVID, but admitted for an alternative reason” or some variation thereof.

- Thank you for this suggestion. We agree that the name we selected was not appropriate for what the group was. We have therefore changed the name of this group to the ‘incidental COVID-19’ group.

5. 29 patients were PCR negative for COVID, yet included in this analysis. This needs to be further clarified in the methods.

- In our data collection section, we explain this with the following statement: ‘’*Patients without molecular confirmation of SARS-CoV2 infection with signs and symptoms consistent with COVID-19 disease based on the case definition in national guidelines and in the absence of a more likely diagnosis were categorized as “clinical COVID-19”.’’*

6. The conclusion needs to be reframed to reflect the study results presented. I agree that the resource limitation issue is very interesting, but it is not the core study question and this study doesn’t adequately assess resource limitations as currently structured.

- Thank you for this suggestion. We have now restructured the Discussions to place more emphasis on the clinical findings (see response to Comment 2 above) and our conclusion in the abstract and in the main text) has been reframed to focus on the core study question. The abstract conclusion reads as follows: ‘’*Clinical COVID-19 care needs to pay careful attention to patient monitoring for complications and management comorbidities. This will require addressing the critical health workforce shortage issues. Prevention of COVID-19 including vaccination particularly among individuals with comorbidities remains a high priority.*

The Conclusion in the paper has also been reworded as follows*: In conclusion, successful management of COVID-19 needs to include evidence-based treatment, close monitoring, and prompt management of clinical complications. This in turn will require addressing health system constraints including health care worker shortages. Prevention of COVID19 including vaccination, particularly among those with comorbidities, should be emphasized.’’*

***Minor***

1) Analyzing clinical characteristics is most useful in assessing risk of admission, complications or death. This paper only examines clinical characteristics of patients who are admitted, limiting any analysis to explain risk for hospitalization without a comparison to the general population. Doing this analysis would require additional investigation, but would be useful to front line clinicians in understanding risk for hospitalization and death (and hence need for higher level of care).

- We thank the reviewer for this suggestion. Our aim was to present the clinical context within hospitalised individuals. Notably, our experience is that there are multifactorial reasons beyond clinical condition, including socioeconomic status, ability to access transport, knowledge of clinical COVID services, etc. that would determine whether individuals with COVID19 sought care. We agree this is an important aspect to study but was beyond the scope of this study.

2) Characteristics of admitted patients (table 1). How was CVD identified? Patient reported history? How was CKD differentiated by AKI? I imagine that prior records were not available for many patients. I would suggest reframing these clinical characteristics to those present at admission rather than historical data. This will provide a better baseline for clinicians who will use this data to assess risk of decompensation among the patients admitted to their service.

- Comorbidities were identified through a check of patient-held records, which are carried by individuals to every consultation with a health provider. These record the diagnosis and plan including medicine prescribed at every contact with health services and therefore provide a record of any existing morbidity. All individuals were asked about specific comorbidities including cardiovascular disease (CVD), diabetes, hypertension, HIV and respiratory disease, and confirmed by the patient-held records, checking against a record of the patient’s drug history. We also sought to confirm these from any accompanying family members. Chronic Kidney Disease (CKD) was differentiated from Acute Kidney Injury (AKI), based on whether there was a pre-existing record of renal disease, including abnormal blood tests. If none, AKI was assumed.

3) Hyperglycemia as a complication is problematic. Agree that tight glucose control is important in critically ill patients, but not certain how useful this finding is in the context of medication induced hyperglycemia. Did all patients get dexamethasone?

- We think it is important to show how many patients experienced hyperglycaemia in our cohort to give clinicians an understanding of how common it was as a complication, regardless of underlying driver (either a known diabetic or a new diagnosis, physiological stress from a severe infection or treatment-induced) - and to draw attention to the need for careful blood glucose monitoring in patients- sometimes neglected in patient care in our setting.
- Not all patients received dexamethasone. In table 2 we show how many patients received Dexamethasone (406/554, 72.1%).

4) Consider additional bins for WBC and Creatinine measurements. Normal vs. not normal is unlikely to be a clinically useful discriminatory tool.

- Thank you for this suggestion. We did have more categories in our analysis, as suggested, but this did not yield any additional clinically useful information and we have therefore used a binary category for clarity.
- We have now added the definitions of ‘high’ and ‘low’ into table 1.

5) The beginning of the fourth paragraph in the discussion section belongs in results, not discussion.

- These results were also shown in the results section and are summarised in the discussion as a reminder and to lead into the discussion about delayed presentation. We have reworded the statement to avoid complete repetition, as follows: ‘’*More than a third of patients presented with severe hypoxia (oxygen saturations below 80%), indicating delayed presentation.’’*

6) The fifth paragraph of the discussion, looking at the frequency of complications, would provide a nice opportunity to compare to other studies in both HIC and LMIC.

- Thank you for this suggestion. We have now added the following to the manuscript: ‘*’Our study showed similar incidences of complications to studies performed in high and middle-income countries.*’’

**Reviewer Two**

**Initial Comments**

***Major***

1) Why not using Cox regression, instead of Poison regression, to asses hospital mortality?

- We used Poisson regression because the outcome was the count of events, rather than the time to event.

2) Only 388 of 563 patients reported a positive PCR test/antigen test for SARS-COV-2. In this sense, please, describe, in methods section, the reasons and criteria used to classify participants with a negative or unknown/ inconclusive PCR/ antigen test result as COVID-19 patients.

- In our data collection section, we explain this with the following statement: ‘Patients without molecular confirmation of SARS-CoV2 infection with signs and symptoms consistent with COVID-19 disease, based on the case definition in national guidelines, in the absence of a more likely diagnosis were categorized as “clinical COVID-19*.’’*

***Minor***

1) Please include the study design in the title.

- This has now been added to our title.

2) Abstract: please include risk ratios with 95% CIs and p-values for the following comparison: "Mortality was higher in those with ≥1 comorbidity than in those without any (43.2% vs 28.2%)".

- The risk ratio and 95% CIs and p-value have been added. The sample has also been reduced to only those who stayed in the ward for more than one night, which aligns with the results presented in the main paper*.*

3) Methods - Study Design and setting: Please describe the number of hospital beds as well as the number of ICU beds (total and COVID-19 dedicated). Some information regarding structure/capacity to provide ventilatory support (eg, oxygen and mechanical ventilation) and health staff (eg patient/physician ratio and nurse/physician ratio) would facilitate the understanding of the local context.

- Thank you for this suggestion-we have now added more detail in Methods as follows:

*‘**A dedicated COVID-19 inpatient centre was established in June 2020 including 3 medical wards with a total of 90 beds with piped oxygen availability, as well as a critical care ward with four beds, with facilities for invasive ventilation. There had been intermittent periods of industrial action by healthcare workers over remunerations, working conditions, and a critical shortage of medical equipment and supplies in the two years preceding the pandemic. Doctors and nurses had taken industrial action over working conditions including the lack of personal protective equipment (PPE) in 2020 disrupting clinical services including provision of haemodialysis. Staff partially returned to work in September 2020. Due to the industrial action during the first COVID19 wave, volunteers were recruited by the hospital to provide clinical care and therefore patient to physician/nurse ratios were variable but significantly more limited than usual. Furthermore, critical care nursing staff and medical personnel to manage ventilation were not available in the first wave.’’*

4) Ethical approval: please describe the number of IRB approval;

- Ethical approval was granted by the Medical Research Council of Zimbabwe (MRCZ). The MRCZ approval ID is MRCZ/E/292. This has now been added to the paper in the ‘Ethical Approval’ section.

5) Tables format: please variable definitions in footnotes (eg, weight category - table 1)

- For weight we classified patients into 3 groups (underweight, normal, and overweight) and as mentioned in our methods section this was done by a visual assessment. *‘’A considerable number of patients were too unwell for measurement of height and weight. Therefore, weight categories were based on a visual assessment by the clinical team. ‘’*

6) Table 1: I might suggest to include p-values for the comparisons between first and second waves;

- Thank you for this suggestion. We have not done this, because there was no expectation that the patients from the two waves would represent the same population, so the null hypothesis is not valid.

7) Discussion - limitations: The authors listed the retrospective data collection as a limitation, but the study is prospective, please clarify. I would add the fact that some important variables that could be associated with outcomes were not measured (eg corticosteroid use).

- We apologise for the confusion this causes. The study was prospective in design, with data collected as patients were admitted. However, the study was conducted in the midst of COVID-19 waves in a routine clinical service. On occasions when it became very busy on the wards, data was entered later e.g. after the patient was discharged, confirming from the medical notes. to ensure all patients were included. We have reworded our statement as follows, removing the term retrospective, as we appreciate this causes confusion. *“The study was conducted during the first two COVID19 waves within a very busy and understaffed clinical service. Therefore, some data are missing and could not be retrospectively obtained from medical notes.”*

**Second round of comments**

’The authors addressed some reviewers comments on this revised manuscript version. Although the manuscript improved, some limitations still need to be addressed.’’

1) Since the authors use mortality as main outcome. I recommend to use a more accurate statistical test (eg, Cox regression) to verify consistency. It could be performed as main analysis or as a complementary sensitivity analysis.

- Thank you for highlighting this. We performed Cox regression as a complementary sensitivity analysis. We have added the following in the statistical analysis section: ‘’As a sensitivity analysis, Cox regression with robust standard errors was used to estimate the mortality hazard ratio in patients who stayed more than 1 night, adjusting for the same variables used in the Poisson multivariate regression.’’
- We have also added in Table 4 (Factors associated with mortality in participants who stayed in the ward for more than 1 night, using multivariate Cox regression models) adding the following into the manuscript ‘’ Table 4 shows the Cox regression for mortality hazard ratio using the same covariates. The association of age category with mortality hazard was not statistically significant on a Wald test. Otherwise, results were comparable to the results of the Poisson regression.’’

2) There are some inconsistencies in manuscript writing. In this sense, I might suggest to edit the manuscript for grammar and style.

- We have been through and reviewed the manuscript to ensure there are no further grammatical errors and to ensure a consistent style throughout. We have made some minor changes to sentence structure to make a more cohesive, easy-flowing paper.

3) Have the authors made all data underlying the findings in their manuscript fully available (please refer to the Data Availability Statement at the start of the manuscript PDF file)?

Reviewer #2 ‘No’

- After discussion with our data team and repository manager as informed consent was not obtained from participants, public deposition of the dataset would breach the Medical Research Council of Zimbabwe (MRCZ) requirements for data from a public hospital. Interested parties should contact the Parirenyatwa Group of Hospitals regarding access to the data. We therefore request an exemption from the editors for the reason mentioned above.

**Reviewer Three**

1) The discussion around the resource limitations in which this service operated are key to be able to lobby for additional health care resources in the future.

   Specifically the statement: "After 1st November 2020, lack of resources was felt to have contributed to 57 (35.4%) of 161 deaths.’’ I feel should be qualified to be "After 1st November 2020, lack of resources was felt by the treating clinical team to have contributed to 57 (35.4%) of 161 deaths."

- We agree the suggested wording more accurately describes it therefore we have changed the statement as guided by the reviewer.

2) Rest of the suggestions are minor and stylistic. See attached comments in PDF. The paper reads well to me and as with all such studies the context is important, and I feel adequately described given the challenges of healthcare delivery in Africa.

- We thank this reviewer for going through the paper and suggesting changes. We have reviewed them all and have made the appropriate changes suggested.

We look forward to hearing from you

Yours sincerely

Dr Arun Fryatt, on behalf of authors
